# Associations of blood trace elements with bone mineral density: a population-based study in US adults

**DOI:** 10.1186/s13018-023-04329-9

**Published:** 2023-11-03

**Authors:** Chunli Wu, Yao Xiao, Yuexia Jiang

**Affiliations:** 1 Xiangya School of Nursing, Central South University, Changsha, 410008 Hunan China; 2 Department of Endocrinology, Endocrinology Research Center, Xiangya Hospital of Central South University, Changsha, 410008 Hunan China; 3 Department of Endocrinology and Metabolism, Guiqian International General Hospital, Guiyang, 550004 China

**Keywords:** Blood trace elements, Lead, Bone mineral density, Osteoporosis

## Abstract

**Background:**

This study aimed to investigate the association between blood trace elements and bone mineral density (BMD) and to determine the association between blood trace elements and the risk of low BMD/osteoporosis among US adults.

**Methods:**

We performed a cross-sectional study using data from National Health and Nutrition Examination Survey (NHANES, 2011–2016). Multivariable linear regression models were employed to assess the associations of BMD in lumbar spine (LS-BMD), pelvic (PV-BMD) and total femur (TF-BMD) with blood trace elements, including Fe, Zn, Cu, Se, Mn, Cd, Pb, Hg. Additionally, the associations of low BMD/osteoporosis with blood trace elements were also evaluated using multivariable logistic regression.

**Results:**

Higher blood Pb levels were found associated with decreased LS-BMD (*p* for trend < 0.001), PV-BMD (*p* for trend = 0.007), and TF-BMD (*p* for trend = 0.003) in female, while higher blood Se levels were associated with increased PV-BMD in female (*p* for trend = 0.042); no linear association between BMD and other blood trace element was observed. Also, significant associations were found between Pb levels and the prevalence of low BMD (*p* for trend = 0.030) and the prevalence of osteoporosis (*p* for trend = 0.036), while association between other blood trace elements and low BMD/osteoporosis was not observed.

**Conclusion:**

This study provides comprehensive insight into the association between blood trace elements and BMD and supports a detrimental effect of blood Pb levels on bone mass in women. Considering our analysis from a representative US general population, further study is warranted for the extreme levels of blood trace elements on bone metabolism.

## Introduction

Osteoporosis has been believed to be a systemic skeletal disease characterized by the loss of bone mass and the degeneration of bone microstructure, resulting in more fragile and brittle bones, and a higher risk of fractures in comparison with normal bones [1]. Osteoporosis has become a major public health problem with the growing elderly population in the world [2]. In 2019, the prevalence of osteoporosis was estimated at 22.1% and 6.6% for women and men aged over 50 in European population, respectively [3]. Fragility fracture incidence also increases with age both in men and women, especially after age of 70 years for women and after age 80 for men [4]. Primary osteoporosis mainly includes postmenopausal osteoporosis and senile osteoporosis. Systemic diseases, endocrine diseases, and malignant neoplasms are among the diseases that cause secondary osteoporosis. Risk factors for osteoporosis are divided into two categories: modifiable and non-modifiable. Weight, smoking, alcohol consumption, low calcium intake, physical inactivity, deficiency of trace elements and vitamins, and long-term glucocorticoid use are among the risk factors for the modifiable osteoporosis group. Gender, age, race, and genetic characteristics are among the risk factors for the non-modifiable osteoporosis group [5]. However, it remains obscure what the certain etiological factors and pathogenesis of osteoporosis are. It is widely accepted that the malnutrition status of certain minerals, such as calcium and magnesium, plays an important role in the development of osteoporosis. Several studies have also indicated that trace elements are essential for growth and development of skeleton by interacting with bone nutritive substance and involving in normal bone metabolism [6–8]. However, the exact roles of multiple trace elements in the pathogenesis of osteoporosis remain largely unknown, and understanding the relationship between elements and bone mineral density (BMD) can provide valuable evidence for the diagnosis and intervention of osteoporosis. Therefore, the aim of this study was to investigate the association between trace elements and BMD, as well as to determine the association between trace elements and the risk of osteopenia/osteoporosis among adults aged ≥ 20 years by using a representative sample from the National Health and Nutrition Examination Survey.

## Materials and methods

### Study population

All participants in this study were obtained from the National Health and Nutrition Examination Survey (NHANES), which aimed to evaluate the nutrition and health status of general United States (US) residents and was based on a cross-sectional design. It collects data that can best or only be obtained by direct physical examination, clinical and laboratory tests, personal interviews, and related measurement procedures. NHANES data are widely used to estimate the prevalence of diagnosed and undiagnosed disease, including acute and chronic conditions, nutritional intake and status, chemical exposures, and various other health-related factors [9].

**Fig. 1:**
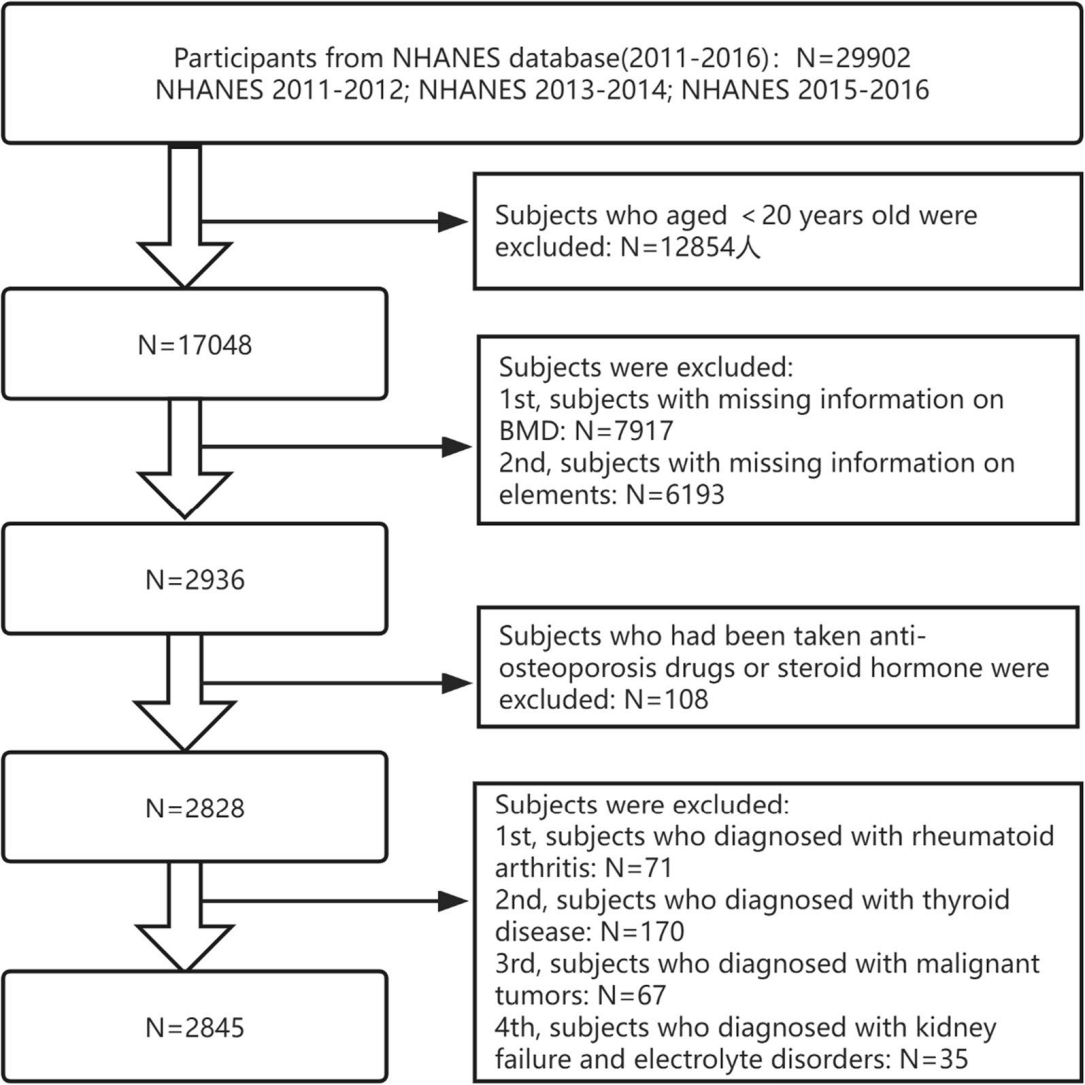
Flow diagram of inclusion criteria and exclusion criteria

In the present study, we extracted data from the NHANES 2011–2016 (2011–2012, 2013–2014, and 2015–2016). The inclusion criteria for participants were as follows: (a) participants with complete BMD and blood trace elements data, (b) participants with complete record of covariates information, (c) participants aged ≥ 20 years. The exclusion criteria were as follows: (I) participants who had a history of female hormone use or glucocorticoid use, (II) participants who were diagnosed with rheumatoid arthritis (RA) by doctors, (III) participants who were diagnosed with cancer by doctors, (IV) participants who diagnosed with thyroid disease by doctors, and (V) participants who diagnosed with renal failure or electrolyte disorders (including hypercalcemia, hypophosphatemia, hypokalemia) by doctors. The detailed process of inclusion and exclusion is shown in Fig. 1. All individuals included in this study provided informed consent, and the ethics review board of the National Center for Health Statistics approved the study.

### BMD testing and low BMD

All participants (included in the final analysis) underwent BMD testing using dual-energy X-ray absorptiometry (DXA) examinations, which were conducted by certified radiology technologists using Hologic QDR-4500A fan-beam densitometers (Hologic; Bedford, MA, USA). All DXA examination data were analyzed using Hologic APEX software (version 4.0). Further details regarding the examination procedures are provided on the NHANES website. Participants who met any of the following criteria were excluded from the DXA examination: pregnancy, self-reported history of radiographic contrast material use in past 7 days, measured weight over 450 pounds, had fractured both hips, had replacements of both hips, or had pins in both hips. In this study, lumbar spine BMD (LS-BMD), pelvic BMD (PV-BMD), and total femur BMD (TF-BMD) were extracted for final analysis.

According to the guidelines of the World Health Organization (WHO), osteoporosis was defined as individuals with BMD values of 2.5 standard deviations (SDs) or more below the mean of the young adult reference group, individuals with all BMD values of 1.0 standard deviations or more above the norm were considered normal BMD, and other cases were considered osteopenia [10]. Finally, we collectively referred to subjects with osteoporosis or osteopenia as having a low BMD.

### Covariates

Considering the potential impact of other factors on bone metabolism, this study also included covariates in the analysis. The selection of covariates available in the NHANES database was based on previous studies [11, 12]. Finally, age, race, education level, body mass index (BMI), smoke status, alcohol consumption, diabetes, hypertension, dyslipidemia, blood calcium, blood phosphorus and serum 25-hydroxyvitamin D_3_[25(OH)D_3_] were considered to be potential confounders in the present study.

### Statistical analysis

Analysis was conducted according to Centers for Disease Control and Prevention (CDC) analytic guidelines for NHANES data, and sample weights were considered for the complex survey design. Data were analyzed using R software (version 4.2.2), IBM SPSS Statistics version 25 (IBM, Armonk, New York, USA) and GraphPad Prism version 9.3.0 (GraphPad Software, San Diego, California, USA), and all reported probabilities (*p* values) were two-sided with *p* < 0.05 considered statistically significant. Means with standard deviation (SD) were used for continuous characteristic variables, and categorical variables were expressed as percentages or frequencies. We used Student's t test for continuous variables and the Chi-square test for categorical variables. Blood trace elements levels were classified according to quartiles (quartile 1: < 25th percentile, quartile 2:25th–50th percentile, quartile 3:50th–75th percentile, quartile 4: > 75th percentile), and multivariable linear regression analysis was performed. Blood trace elements were evaluated by using quartile 1 as the reference. In multivariate linear regression models, age, race, BMI, smoking status, alcohol consumption, status of hypertension, status of diabetes, status of hypertension, status of dyslipidemia, blood calcium levels, blood phosphorus levels, blood 25-OHD_3_ were adjusted. Furthermore, multivariable logistic regression was used to evaluate the risk of low BMD/osteoporosis and blood trace elements.

## Result

### Characteristics of participants

**Fig. 2:**
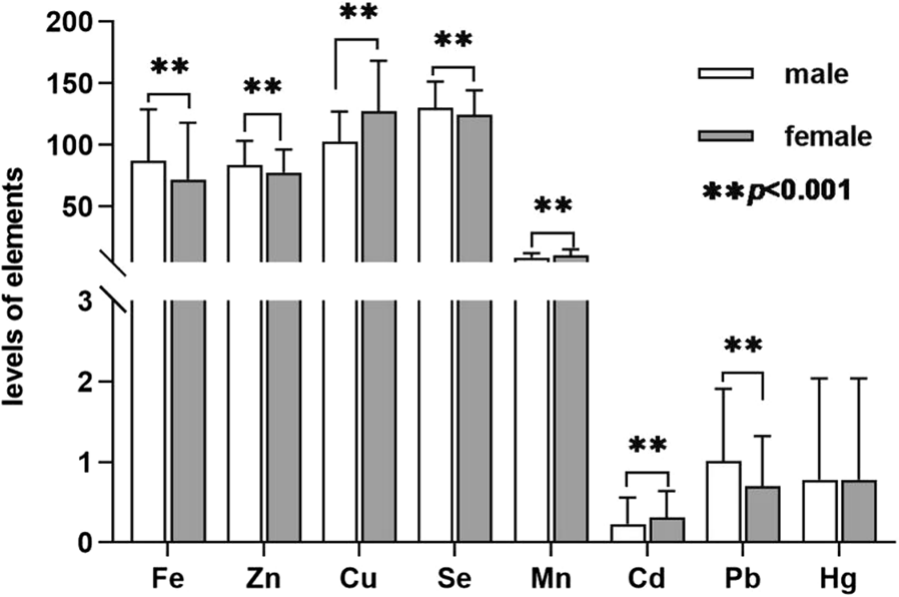
Blood trace element levels in different genders. Fe, iron, (ug/dL); Zn, zinc, (ug/dL); Cu, copper, (ug/dL); Se, selenium, (ug/L); Mn, manganese, (ug/L); Cd, cadmium, (ug/L); Pb, lead, (ug/dL); Hg, mercury, (ug/L)

The baseline characteristics of study participants are listed in Table 1. All participants (1402 males, 1083 females) included in the analysis had a mean age of 37.98 ± 11.25 years. The mean LS-BMD, PV-BMD and TF-BMD were 1.036 ± 0.154, 1.252 ± 0.171, 1.185 ± 0.140, respectively. Compared with female, male tended to have higher prevalence of smoking, alcohol consumption, hypertension, diabetes, and dyslipidemia. Meanwhile, PV-BMD and TF-BMD in male were significantly higher than female. In contrast, females usually have higher prevalence of obese. Moreover, we compared blood trace elements levels among different gender. The results showed that blood levels of iron, zinc, selenium, and lead were higher in males (*p* < 0.001), while blood levels of copper, manganese and cadmium were higher in female (*p* < 0.001), and there was no significant difference found in blood mercury level between different gender. The results are listed in Fig. 2.

**Table 1:** Baseline characteristics of unweighted participants included in the analysis

Characteristic	Total (*n* = 2485)	Male (*n* = 1402, 56.4%)	Female (*n* = 1083, 43.6%)	*p-value*
Age (years)	37.98 ± 11.25	38.27 ± 11.31	37.60 ± 11.18	0.149
Race, *n* (%)				**0.021**
Mexican American	382(15.4%)	193(13.8%)	189(17.5%)	
Other Hispanic	271(10.9%)	140(10.0%)	131(12.1%)	
Non-Hispanic white	828(33.3%)	490(35.0%)	338(31.2%)	
Non-Hispanic black	556(22.4%)	318(22.7%)	238(22.0%)	
Other races	448(18.0%)	261(18.6%)	187(17.3%)	
Education level, *n* (%)				**0.018**
Under high school	382(23.8%)	281(20.0%)	186(17.2%)	
High school or equivalent	256(48.7%)	745(53.1%)	567(52.4%)	
Above high school	145(27.6%)	376(26.8%)	330(30.5%)	
BMI kg/m^2^ *n* (%)				0.336
Normal (BMI < 25 kg/m^2^)	807(32.5%)	435(31.0%)	372(34.3%)	
Overweight (25 ≤ BMI < 30 kg/m^2^)	777(31.3%)	497(25.4%)	280(25.9%)	
Obese (BMI ≥ 30 kg/m^2^)	901(36.3%)	470(33.5%)	431(39.8%)	
Smoke status, *n* (%)				** < 0.001**
No	1537(61.9%)	758(54.1%)	779(71.9%)	
Yes	948(38.1%)	644(45.9%)	304(28.1%)	
Alcohol consumption, *n* (%)				** < 0.001**
No	715(28.8%)	284(20.3%)	431(39.8%)	
Yes	1770(71.2%)	1118(79.7%)	652(60.2%)	
Hypertension, *n* (%)				** < 0.001**
No	2259(90.9%)	1249(89.1%)	1010(93.3%)	
Yes	226(9.1%)	153(10.9%)	73(6.7%)	
Diabetes, *n* (%)				0.161
No	2328(93.7%)	1305(93.1%)	1023(94.5%)	
Yes	157(6.3%)	97(6.9%)	60(5.5%)	
Dyslipidemia, *n* (%)				** < 0.001**
No	1760(70.8%)	885(63.1%)	875(80.8%)	
Yes	725(29.2%)	517(36.9%)	208(19.2%)	
Blood calcium (mmol/L)	2.35 ± 0.08	2.36 ± 0.80	2.33 ± 0.82	** < 0.001**
Blood phosphorus (mmol/L)	1.21 ± 0.18	1.21 ± 0.18	1.22 ± 0.18	**0.020**
Blood 25-OHD_3_ (nmol/L)	55.52 ± 23.57	55.14 ± 21.78	56.03 ± 25.70	0.863
LS-BMD (g/cm^2^)	1.036 ± 0.154	1.033 ± 0.163	1.041 ± 0.142	0.219
PV-BMD (g/cm^2^)	1.252 ± 0.171	1.277 ± 0.178	1.220 ± 0.156	** < 0.001**
TF-BMD (g/cm^2^)	1.185 ± 0.140	1.242 ± 0.133	1.111 ± 0.111	** < 0.001**

Continuous variables were presented by mean ± standard deviation (SD), and categorical variables were presented with numbers and percentages (%). T-test was used to assess the statistical difference and values in boldface are significantly different (*p* < 0.05) among different genders

BMI, body mass index; 25(OH)D_3_, 25-hydroxyvitamin D_3_, BMD, bone mineral density; LS, lumbar spine; PV, Pelvis; TF, Total femur

### Association between blood trace elements and BMD

Table 2 presents the results of the multivariate linear regression analysis examining the association between blood trace elements levels and BMD. The associations between blood trace elements and BMD in different bone sites were also separately explored among the study population (Table 2). In multivariate linear regression models, increased blood Pb levels were found to be associated with LS-BMD (*p* for trend < 0.001), PV-BMD (*p* for trend = 0.007), and TF-BMD (*p* for trend = 0.003) in women. Women with high level of blood Pb (Q4) have a lower LS-BMD (β-coefficient = -0.065), lower PV-BMD (β-coefficient = − 0.040), and a lower TF-BMD (β-coefficient = − 0.090). In addition, increased blood Se level was found to be associated with PV-BMD in women (*p* for trend = 0.042), but the effect was not significant when comparing the group with highest level of blood Se (Q4) with the lowest group (Q1). Further, trend association between other blood trace elements and BMD was not observed (*p* for trend > 0.05). In quartile analyses, compared with the lowest level, positive associations were observed between blood Fe level Q3 and LS-BMD (β coefficient = 0.029), positive association was observed between blood level Q2 and PV-BMD (β coefficient = 0.034). Also, significant positive association was observed in men blood Zn levels and PV-BMD (β coefficient = 0.029,), men blood Zn level Q4 with TF-BMD (β coefficient = 0.045).

**Table 2:** Multivariate linear regression analysis for blood trace elements and BMD

	LS-BMD (g/cm^2^)		PV-BMD (g/cm^2^)		TF-BMD (g/cm^2^)	
	Male	Female	Male	Female	Male	Female
	*β*(95% CI)	*β*(95% CI)	*β*(95% CI)	*β*(95% CI)	*β*(95% CI)	*β*(95% CI)
*Fe*						
Q1	Reference	Reference	Reference	Reference	Reference	Reference
Q2	0.008 ( − 0.018,0.035)	0.01 ( − 0.016,0.037)	0.01 ( − 0.019,0.039)	**0.034 (0.006,0.062)**	0.021 ( − 0.02,0.063)	0.028 ( − 0.014,0.069)
Q3	− 0.007 ( − 0.04,0.026)	**0.029 (0.001,0.058)**	0.008 ( − 0.034,0.049)	0.021 ( − 0.01,0.053)	0.006 ( − 0.044,0.057)	0.002 ( − 0.041,0.044)
Q4	0.016 ( − 0.011,0.044)	0.006 ( − 0.027,0.04)	0.022 ( − 0.006,0.05)	0.009 ( − 0.023,0.04)	0.025 ( − 0.026,0.075)	0.053 ( − 0.025,0.132)
*p* for trend	0.394	0.361	0.171	0.633	0.553	0.187
*Zn*						
Q1	Reference	Reference	Reference	Reference	Reference	Reference
Q2	0.03 ( − 0.001,0.06)	− 0.014 ( − 0.039,0.012)	**0.038 (0,0.077)**	− 0.017 ( − 0.046,0.013)	0.044 ( − 0.01,0.099)	0.015 ( − 0.035,0.066)
Q3	0.01 ( − 0.015,0.035)	− 0.021 ( − 0.045,0.004)	**0.029 (0.004,0.054)**	− 0.013 ( − 0.045,0.02)	0.031 ( − 0.017,0.078)	− 0.004 ( − 0.052,0.044)
Q4	0.016 ( − 0.01,0.042)	− 0.014 ( − 0.043,0.015)	0.019 ( − 0.009,0.048)	− 0.019 ( − 0.05,0.012)	**0.045 (0,0.09)**	− 0.034 ( − 0.085,0.017)
*p* for trend	0.665	0.196	0.474	0.301	0.091	0.267
*Cu*						
Q1	Reference	Reference	Reference	Reference	Reference	Reference
Q2	− 0.01 ( − 0.034,0.014)	0.008 ( − 0.033,0.049)	− 0.004 ( − 0.03,0.021)	0.018 ( − 0.033,0.068)	− 0.026 ( − 0.061,0.009)	− 0.013 ( − 0.086,0.06)
Q3	0.003 ( − 0.022,0.028)	0.013 ( − 0.031,0.057)	− 0.004 ( − 0.032,0.024)	0.026 ( − 0.018,0.07)	− 0.005 ( − 0.04,0.03)	0.014 ( − 0.063,0.092)
Q4	− 0.012 ( − 0.045,0.021)	0.01 ( − 0.033,0.054)	− 0.041 ( − 0.085,0.003)	0.018 ( − 0.027,0.062)	− 0.036 ( − 0.092,0.021)	− 0.003 ( − 0.062,0.055)
*p* for trend	0.820	0.708	0.143	0.586	0.336	0.931
*Se*						
Q1	Reference	Reference	Reference	Reference	Reference	Reference
Q2	0.005 ( − 0.02,0.031)	− 0.01 ( − 0.04,0.021)	0.008 ( − 0.019,0.036)	0 ( − 0.024,0.024)	0.012 ( − 0.031,0.055)	− 0.002 ( − 0.041,0.037)
Q3	0.008 ( − 0.018,0.033)	0.005 ( − 0.024,0.034)	− 0.015 ( − 0.039,0.009)	0.027 ( − 0.003,0.056)	− 0.006 ( − 0.047,0.034)	0.054 ( − 0.001,0.11)
Q4	0 ( − 0.029,0.029)	0.003 ( − 0.027,0.033)	− 0.015 ( − 0.04,0.009)	0.025 ( − 0.008,0.058)	− 0.003 ( − 0.05,0.043)	0.024 ( − 0.023,0.071)
*p* for trend	0.956	0.632	0.102	0.042	0.687	0.080
*Mn*						
Q1	Reference	Reference	Reference	Reference	Reference	Reference
Q2	0.006 ( − 0.021,0.033)	− 0.007 ( − 0.043,0.029)	0.01 ( − 0.015,0.035)	− 0.01 ( − 0.046,0.025)	0.017 ( − 0.03,0.065)	0.027 ( − 0.064,0.117)
Q3	0.024 ( − 0.007,0.055)	− 0.012 ( − 0.044,0.02)	0.022 ( − 0.008,0.052)	− 0.001 ( − 0.033,0.031)	0.027 ( − 0.012,0.066)	− 0.007 ( − 0.057,0.043)
Q4	− 0.008 ( − 0.04,0.025)	− 0.023 ( − 0.051,0.005)	0.014 ( − 0.027,0.054)	− 0.027 ( − 0.056,0.003)	− 0.014 ( − 0.07,0.042)	− 0.025 ( − 0.066,0.016)
*p* for trend	0.589	0.097	0.246	0.095	0.831	0.095
*Cd*						
Q1	Reference	Reference	Reference	Reference	Reference	Reference
Q2	− 0.002 ( − 0.029,0.025)	0.029 ( − 0.004,0.062)	0.015 ( − 0.014,0.044)	0.02 ( − 0.015,0.054)	0.008 ( − 0.031,0.047)	0.034 ( − 0.01,0.079)
Q3	0.008 ( − 0.023,0.038)	0.02 ( − 0.011,0.052)	0.021 ( − 0.018,0.059)	0.016 ( − 0.017,0.048)	0.03 ( − 0.016,0.076)	0.028 ( − 0.014,0.069)
Q4	0.009 ( − 0.021,0.039)	− 0.007 ( − 0.047,0.034)	0.027 ( − 0.009,0.062)	− 0.014 ( − 0.048,0.02)	0.021 ( − 0.028,0.071)	0.015 ( − 0.04,0.07)
*p* for trend	0.518	0.752	0.119	0.529	0.271	0.617
*Pb*						
Q1	Reference	Reference	Reference	Reference	Reference	Reference
Q2	− 0.009 ( − 0.033,0.015)	− 0.017 ( − 0.047,0.013)	− 0.005 ( − 0.037,0.026)	− 0.007 ( − 0.035,0.02)	0.012 ( − 0.042,0.066)	0.001 ( − 0.038,0.039)
Q3	0.013 ( − 0.015,0.041)	** − 0.032 ( − 0.056, − 0.007)**	0.005 ( − 0.026,0.035)	** − 0.044 ( − 0.077, − 0.012)**	0.036 ( − 0.017,0.089)	− 0.019 ( − 0.083,0.045)
Q4	− 0.008 ( − 0.034,0.018)	** − 0.065 ( − 0.099, − 0.032)**	− 0.003 ( − 0.036,0.029)	** − 0.04 ( − 0.079, − 0.001)**	− 0.004 ( − 0.06,0.052)	** − 0.09 ( − 0.144, − 0.037)**
*p* for trend	0.902	< 0.001	0.990	0.008	0.936	0.003
*Hg*						
Q1	Reference	Reference	Reference	Reference	Reference	Reference
Q2	0.008 ( − 0.012,0.029)	− 0.001 ( − 0.033,0.032)	0.02 ( − 0.007,0.046)	− 0.01 ( − 0.045,0.025)	0.037 ( − 0.006,0.081)	− 0.028 ( − 0.104,0.048)
Q3	0.001 ( − 0.021,0.023)	0.013 ( − 0.013,0.039)	− 0.008 ( − 0.041,0.025)	− 0.004 ( − 0.037,0.029)	− 0.011 ( − 0.05,0.028)	− 0.057 ( − 0.127,0.012)
Q4	0.004 ( − 0.022,0.029)	− 0.02 ( − 0.053,0.013)	− 0.012 ( − 0.041,0.017)	− 0.004 ( − 0.042,0.033)	0.035 ( − 0.012,0.083)	− 0.047 ( − 0.133,0.039)
*p* for trend	0.898	0.406	0.249	0.912	0.433	0.188

Multivariate linear regression adjusted for age, race, education level, BMI, smoking status, alcohol consumption, status of hypertension, status of diabetes, status of dyslipidemia, blood calcium levels, blood phosphorus levels, blood 25-OHD3

Values in boldface are significantly different (*p* < 0.05) from the reference group

### Association between blood trace elements and risk of low BMD/osteoporosis

The associations of the risk of low BMD/osteoporosis with blood trace elements are listed in Table 3. Multivariable logistic regression analysis showed that a striking association for increased blood Pb level with the prevalence of low BMD (*p* for trend = 0.030), and the presence of osteoporosis (*p* for trend = 0.036). At the extreme, women with highest levels of blood Pb have a higher risk of low BMD (OR = 2.060, 95%CI = 1.043–4.069) and osteoporosis (OR = 4.629, 95%CI = 1.026–20.881) compared with the women with the lowest level of blood Pb, respectively. Besides, high level of blood Mn (Q3) was associated with a lower risk of low BMD (OR = 0.614, 95%CI = 0.379–0.995) in male.

**Table 3:** Association between blood trace elements and the risk of low BMD/osteoporosis

Elements		Normal BMD vs low BMD		Non-Osteoporosis vs Osteoporosis	
		*OR*(95%*CI*)	*P for trend*	*OR*(95%*CI*)	*P for trend*
*Male*					
Fe	Q1	1	0.248	1	0.483
	Q2	0.981 (0.612,1.571)		1.359 (0.4,4.611)	
	Q3	1.002 (0.569,1.764)		0.762 (0.222,2.612)	
	Q4	0.765 (0.469,1.249)		0.887 (0.292,2.697)	
Zn	Q1	1	0.950	1	0.707
	Q2	0.81 (0.518,1.267)		0.361 (0.139,0.938)	
	Q3	0.949 (0.613,1.468)		0.759 (0.289,1.996)	
	Q4	0.911 (0.592,1.403)		0.674 (0.318,1.429)	
Cu	Q1	1	0.882	1	0.462
	Q2	1.255 (0.827,1.904)		1.158 (0.557,2.405)	
	Q3	0.939 (0.606,1.452)		0.54 (0.181,1.605)	
	Q4	1.225 (0.621,2.416)		0.823 (0.126,5.375)	
Se	Q1	1	0.185	1	0.828
	Q2	1.019 (0.584,1.778)		0.403 (0.138,1.177)	
	Q3	0.92 (0.588,1.44)		0.689 (0.245,1.938)	
	Q4	1.403 (0.844,2.333)		0.744 (0.277,1.996)	
Mn	Q1	1	0.230	1	0.585
	Q2	0.74 (0.508,1.079)		1.586 (0.523,4.811)	
	Q3	**0.614 (0.379,0.995)**		0.816 (0.323,2.057)	
	Q4	0.946 (0.617,1.451)		0.931 (0.316,2.739)	
Cd	Q1	1	0.746	1	0.807
	Q2	0.988 (0.677,1.442)		1.652 (0.657,4.151)	
	Q3	0.995 (0.628,1.578)		0.841 (0.279,2.53)	
	Q4	0.914 (0.547,1.525)		0.969 (0.325,2.885)	
Pb	Q1	1	0.305	1	0.615
	Q2	1.028 (0.675,1.565)		1.286 (0.349,4.738)	
	Q3	0.783 (0.486,1.26)		0.687 (0.176,2.685)	
	Q4	0.865 (0.551,1.357)		1.402 (0.493,3.988)	
Hg	Q1	1	0.399	1	0.503
	Q2	0.779 (0.517,1.176)		1.227 (0.461,3.265)	
	Q3	0.941 (0.602,1.471)		1.087 (0.354,3.34)	
	Q4	0.765 (0.478,1.225)		0.617 (0.194,1.96)	
*Female*					
Fe	Q1	1	0.736	1	0.206
	Q2	0.961 (0.585,1.578)		2.042 (0.765,5.453)	
	Q3	0.767 (0.472,1.245)		2.648 (0.981,7.153)	
	Q4	1.224 (0.674,2.222)		1.558 (0.429,5.661)	
Zn	Q1	1	0.994	1	0.392
	Q2	1.415 (0.864,2.318)		1.491 (0.511,4.349)	
	Q3	1.252 (0.777,2.015)		1.901 (0.62,5.829)	
	Q4	0.972 (0.552,1.713)		1.534 (0.361,6.521)	
Cu	Q1	1	0.178	1	0.454
	Q2	0.797 (0.459,1.384)		0.615 (0.093,4.072)	
	Q3	0.62 (0.305,1.257)		0.501 (0.099,2.522)	
	Q4	0.608 (0.307,1.206)		0.515 (0.096,2.756)	
Se	Q1	1	0.371	1	0.460
	Q2	1.21 (0.727,2.015)		2.307 (0.779,6.833)	
	Q3	1.041 (0.639,1.696)		1.709 (0.491,5.948)	
	Q4	0.768 (0.425,1.387)		1.69 (0.588,4.856)	
Mn	Q1	1	0.333	1	0.576
	Q2	1.266 (0.607,2.639)		0.931 (0.163,5.301)	
	Q3	1.103 (0.603,2.019)		1.116 (0.281,4.427)	
	Q4	1.388 (0.792,2.433)		1.423 (0.354,5.722)	
Cd	Q1	1	0.104	1	0.738
	Q2	1.056 (0.616,1.812)		0.511 (0.121,2.152)	
	Q3	1.195 (0.676,2.114)		0.374 (0.088,1.577)	
	Q4	1.773 (0.938,3.353)		0.67 (0.126,3.548)	
Pb	Q1	1	0.030	1	0.036
	Q2	1.409 (0.822,2.414)		3.305 (0.664,16.444)	
	Q3	1.461 (0.914,2.337)		3.267 (0.748,14.27)	
	Q4	**2.06 (1.043,4.069)**		**4.629 (1.026,20.881)**	
Hg	Q1	1	0.454	1	0.625
	Q2	0.758 (0.419,1.372)		1.988 (0.386,10.236)	
	Q3	0.663 (0.396,1.11)		1.378 (0.251,7.572)	
	Q4	0.87 (0.515,1.47)		1.912 (0.284,12.87)	

Multivariate logistic regression adjusted for age, race, education level, BMI, smoking status, alcohol consumption, status of hypertension, status of diabetes, status of hypertension, status of dyslipidemia, blood calcium levels, blood phosphorus levels, blood 25-OHD3

Values in boldface are significantly different (*p* < 0.05) from the reference group

## Discussion

The present study explored the correlation between blood trace elements and BMD of lumbar spine, pelvis and total femur in a US population aged ≥ 20 years. Based on the representative sample of the US population in NHANES (2011–2016), we found that higher blood Pb level was associated with lower LS-BMD, PV-BMD, and TF-BMD in female. Also, increased blood Pb level was significantly associated with the prevalence of low BMD/osteoporosis. Besides blood Pb and blood Se, we did not observe any significant linear relationship between other blood trace elements with BMD. Our study suggests that in a representative sample of US population, higher levels of blood Pb have a detrimental effect on bone mass, but difference in other blood trace elements may not be sufficient to affect bone metabolism. However, the effect of extreme levels of blood trace element on bone warrants further investigation.

Lead is a well-known heavy metal toxin that can adversely affect various body systems, including the skeletal system. The negative impact of lead on bone density can be attributed to several mechanisms. Firstly, lead inhibits osteoblast activity by suppressing the key synthesis pathway, Wnt/β-catenin signaling, and induces osteoblast apoptosis [13]. Secondly, lead may interfere with the synthesis of osteocalcin, which promotes bone mineralization, and due to its higher affinity for hydroxyapatite, it can replace calcium in the bone matrix, affecting bone matrix formation [14]. Furthermore, lead indirectly affects calcium-phosphate metabolism by inhibiting renal 1-α hydroxylase, which is closely related to osteoporosis development [15]. Our results suggest that blood lead levels were significantly associated with BMD on the whole. It highlights the importance of considering lead exposure as a potential risk factor for osteoporosis in clinical assessments. Moreover, early detection and intervention to reduce lead exposure may help mitigate the adverse effects on bone health and prevent the progression of osteoporosis. Cadmium and mercury are also heavy metals and important environmental pollutants. Previous study has shown a negative correlation between lumbar spine bone density and blood cadmium levels, with a 0.01 g/cm^2^ decrease in lumbar spine bone density for every 1 μg/L increase in blood cadmium levels, and the subgroup analysis indicated that the negative association between cadmium and bone density was observed only in non-Hispanic Black individuals [16]. Mercury is thought to be released into the atmosphere through industrial emissions and is transformed by water bacteria into methylmercury, which then accumulates in fish. Therefore, the main source of mercury exposure in the population is fish consumption. Studies have shown that higher blood mercury levels are positively correlated with increased femoral neck bone density in Korean men, suggesting a potential protective effect of blood mercury against osteoporosis [17]. A study conducted in the US population between 2008 and 2010 also reported a positive correlation between femoral bone density and blood mercury levels [18]. This relationship may be related to diet, as fish consumption is a major source of mercury in Korean and American populations. However, fish also contain n-3 and n-6 fatty acids, which may also have a preventive effect on osteoporosis. In our study, we did not observe a significant association between blood cadmium and mercury with BMD. This may be due to the low exposure of them in our study sample, and the average concentration of blood cadmium in our study sample is low (median = 0.23, IQR = 0.15–0.48), which may not reflect the effect of high blood cadmium on bone metabolism.

Iron is involved in the synthesis of bone matrix and the activation of 25-hydroxyvitamin D. Iron is also a component of human hemoglobin and myoglobin, involved in the transport and storage of oxygen in the body, and is also involved in the synthesis of catalase and cytochrome oxidase. Wang et al. [19] found a significant increase in iron levels in elderly men with osteoporosis compared to the healthy group, and a negative association between iron and bone mineral density, suggesting that excess iron in serum may contribute to bone loss. Sung-Min Kim et al. [20] investigated the association between lumbar spine BMD and serum iron levels of premenopausal women in Korea; result showed a significant negative correlation between lumbar spine BMD and serum iron levels. An epidemiological survey of 290 postmenopausal women aged 45–65 years in Xi'an showed no significant differences in trace elements between the osteoporosis, osteopenia, and healthy groups, nor was a significant correlation observed between serum iron and BMD [21]. The present study yielded similar results that iron levels in men were significantly higher, but there were no significant differences between the groups with different bone mass levels (normal bone density, low bone mass and osteoporosis), while the osteoporotic group, female had higher iron levels than the low bone mass and normal bone density groups. After transforming iron into a categorical variable based on percentile, no significant association was found between iron and BMD, nor was there a correlation between iron and the prevalence of low BMD/osteoporosis. It has been reported that estrogen can regulate the metabolism of trace elements in the body directly or indirectly [21], and the decrease in estrogen levels after menopause leads to an increase in iron accumulation [22, 23]. In the present study, the majority of the osteoporosis group was over 50 years; we speculate that the decline in estrogen levels may have potentially affected the homeostasis of trace elements. In contrast to Sung-Min Kim's results, we did not find a significant association between iron and BMD. This discrepancy might be related to differences in study populations or iron exposure levels. The subjects in this study were American and had a slightly lower mean serum iron concentration of 85.16 ug/dL.

Zinc is an activator of many metalloenzymes and is also involved in bone metabolism. Zinc induces the activity of bone metabolic enzymes such as alkaline phosphatase, collagenase and sulfatase and also affects the levels of 1, 25-OHVD_3_ and calcitonin [24]. On the one hand, zinc stimulates osteoblast differentiation and also directly activates aminoacyl transfer RNA synthetase in osteoblasts and stimulates collagen synthesis; on the other hand, zinc also inhibits osteoclast formation and stimulates apoptosis of osteoclasts in the process of bone resorption [25]. Deniz et al. [26] observed that blood zinc was positively associated with lumbar spine bone mineral density (total T score) among Turkey postmenopausal women, but no significant differences were found in the healthy, low bone mass and osteoporosis groups. This positive relationship was found in men in the present study but not in women. In our study, men with higher blood zinc levels have a high PV-BMD and TF-BMD than men with the lowest levels of blood zinc, but the linear trend was not significant. In women, a lower zinc was negatively associated with pelvic bone mineral density. The potential mechanisms underlying may be lower blood zinc levels in women. It has been suggested that zinc deficiency may indirectly affect bone mineral density by reducing intestinal calcium absorption and enhance the bone resorption effects of PTH through increasing PTH levels [27]. Zinc deficiency has also been shown to reduce osteoblast activity, decrease collagen, proteoglycan synthesis, and impede bone salt deposition [28].

Copper plays a key role in the cross-linking of collagen and elastin, an essential component of the bone matrix, the integrity of which is important for the strength and plastic deformation of bone [29]. In addition, copper positively affects the proliferation and function of osteoblasts and indirectly promotes osteogenic and lipogenic differentiation of bone marrow mesenchymal stem cells (BMSCs) [30]. Several previous studies have confirmed the association between copper and bone health; among these studies, the association between Cu and bone mass has led to conflicting conclusions. A study of 722 participants found that total femur density and femoral neck bone mineral density in the group with the lowest serum copper concentration (< 98.5 ug/dL) were lower than those in the group with the second concentration (98.5–114 ug/dL), and those with the highest serum copper concentration (≥ 134 ug/dL) were lower than those in the group with the second concentration. The risk of a total fracture increased approximately fourfold. In addition, there was a significant linear association between serum copper levels and total fractures in men. This suggests that low levels of serum copper led to decreased BMD, while higher serum copper levels increase fracture risk [31]. Okyay et al. [32] found that compared with postmenopausal women without osteoporosis, the serum copper level was lower in early postmenopausal women with osteoporosis, and there was a strong correlation between low serum copper level and low bone mineral density in the total femur, femoral neck and lumbar vertebrae. A meta-analysis of serum trace elements and osteoporosis suggested that low serum levels of serum iron, zinc, and copper seemed to be important risk factors for osteoporosis [33]. The mechanisms of how copper affects bone density are not fully understood; the possible mechanisms are as follows: firstly, as a co-factor of antioxidant enzymes, copper can affect the function of bone cells by regulating the activity of antioxidant enzymes, such as lysine oxidase, a necessary enzyme participated in covalent cross-linking of collagen [34]; secondly, copper not only stimulates the differentiation of bone marrow mesenchymal stem cells into osteoblasts, but also promotes the growth and activity of osteoblasts [30]; thirdly, copper induces a hypoxic microenvironment by promoting the expression of bone-related genes, thereby inhibiting the process of bone resorption, and promote angiogenesis through enhancing the expression of vascular endothelial growth factor [35].

Selenium acts in bone health through specific selenoproteins, which have antioxidant enzyme activity and are responsible for maintaining cellular redox homeostasis, regulating inflammation, osteoblast proliferation/differentiation [36]. Oxidative stress leads to excessive apoptosis of bone cells, which breaks the balance between bone resorption and bone formation, thereby increasing the bone remodeling process and bone mass loss, while selenium protein protects bone cells from oxidative stress damage in the bone microenvironment through its antioxidant activity [37]. Mutations in the selenoprotein gene and reduced circulating selenium levels have been found to be associated with bone disease, and selenium intake appears to be inversely associated with the risk of fragility fractures of the hip [38]. Recent studies have observed that altered selenium status may also affect bone mass, with higher concentrations of selenoprotein, and selenium was positively associated with higher total femur and rotor site BMD in men [39]. Most published studies on selenium and bone mineral density have reported a protective effect of selenium on bones, but selenium is not always positive in humans; adverse health effects have been observed when selenium levels higher than 90–120 ug/L in some observational studies and clinical trials [40, 41]. The recommended dietary intake of selenium is 55 μg/L per day, and in selenium-rich populations, selenium intake above the recommended amount does not increase selenoprotein viability or activity, but rather increases plasma selenium concentrations [90]. A few studies have reported that serum selenium level and dietary selenium intake are not associated with osteoporosis [19, 42]. The study results of Marta Galvez-Fernandez et al. [43] showed that the association between selenium and bone mineral density presented a nonlinear dose response, with a negative correlation below 105 μg/L and a positive correlation above 105 ug/L, and there was no significant correlation when age and sex were subgroup-analyzed. A cross-sectional study assessing plasma selenium and oxidative stress levels observed a nonlinear dose-dependent relationship between selenium exposure and a biomarker of oxidative stress (8-oxo-dG), with a positive correlation between blood selenium and 8-oxo-dG when plasma selenium concentrations were greater than 110 ug/L [44].

Manganese is a component of various enzymes involved in cartilage ossification and mucopolysaccharide synthesis. Bone diseases caused by manganese deficiency are directly caused by defects in the enzymes of glycosaminoglycan synthesis, and excess manganese may interfere with the metabolism of other elements. Compared with previous studies, our results showed that bone mineral density of lumbar spine, pelvis and total femur were negatively correlated with high levels of manganese. Subgroup analysis showed that blood manganese was negatively correlated with bone mineral density of three sites aged 20–30 years or with normal BMI. There was no significant correlation between lumbar bone density and other races. In women, the negative correlation between blood manganese and bone mineral density was mainly found in individuals aged 20–50 years or overweight. The results of a cross-sectional study showed that blood manganese was negatively correlated with bone mineral density and bone mineral in the femoral neck and the whole body, especially in the femoral neck bone mineral density of women aged 50–70 years [45]. A cross-sectional study including 304 retired workers with occupational manganese exposure assessed the relationship between long-term occupational manganese exposure and bone mass and found that female participants in the highest manganese exposure group had significantly lower levels of stiffness index (SI) and T-score than controls [46], suggesting that women with occupational manganese exposure are at higher risk of osteoporosis. Our study draws similar conclusion; therefore, people in long-term manganese-exposed occupations should be alert to the development of osteoporosis and check bone density regularly.

Although this study provides reference value for clinical practice and future research, there are still several limitations. First, our study is a cross-sectional analysis and no causal inference between blood trace elements and bone mineral density can be made. Second, although some covariates like demographic, medical history, etc., were adjusted, confounding variables such as diet and lifestyle not included in this analysis may have underlying affected the correction between blood trace elements and bone mineral density.

## Conclusion

In conclusion, our study presents a comprehensive insight into the spectrum of the effect of blood trace elements on BMD. Our results demonstrated that blood Pb levels have detrimental effect on the LS-BMD, PV-BMD, and TF-BMD, while no significant association between other blood trace elements with BMD was observed. However, given the inherent limitations of the present study, further studies are needed to verify these findings and explore the underlying biological mechanism.

## Data Availability

Publicly available datasets were analyzed in this study. This data can be found here: https://www.cdc.gov/nchs/nhanes/.
